# Blood Pressure in the First 6 Hours Following Endovascular Treatment for Ischemic Stroke Is Associated With Outcome

**DOI:** 10.1161/STROKEAHA.120.033657

**Published:** 2021-09-20

**Authors:** Noor Samuels, Rob A. van de Graaf, Carlijn A.L. van den Berg, Simone M. Uniken Venema, Kujtesa Bala, Pieter Jan van Doormaal, Wouter van der Steen, Elbert Witvoet, Jelis Boiten, Heleen den Hertog, Wouter J. Schonewille, Jeannette Hofmeijer, Floris Schreuder, Tobien A.H.C.M.L. Schreuder, H. Bart van der Worp, Yvo B.W.E.M. Roos, Charles B.L.M. Majoie, James F. Burke, Adriaan C.G.M. van Es, Aad van der Lugt, Bob Roozenbeek, Hester F. Lingsma, Diederik W.J. Dippel

**Affiliations:** Department of Neurology (N.S., R.A.v.d.G., K.B., W.v.d.S., B.R., D.W.J.D.), Erasmus MC University Medical Center, Rotterdam, the Netherlands.; Department of Radiology and Nuclear Medicine (N.S., R.A.v.d.G., C.A.L.v.d.B., P.J.v.D., W.v.d.S., A.v.d.L., B.R.), Erasmus MC University Medical Center, Rotterdam, the Netherlands.; Department of Public Health (N.S., H.F.L.), Erasmus MC University Medical Center, Rotterdam, the Netherlands.; Department of Neurology and Neurosurgery, Brain Center, University Medical Center Utrecht, the Netherlands (S.M.U.V., H.B.v.d.W.).; Department of Neurology, Haga Hospital, Den Haag, the Netherlands (E.W.).; Department of Neurology, Haaglanden Medical Center, Den Haag, the Netherlands (J.B.).; Department of Neurology, Isala Hospital, Zwolle, the Netherlands (H.d.H.).; Department of Neurology, Sint Antonius Hospital, Nieuwegein, the Netherlands (W.J.S.).; Department of Neurology, Rijnstate Hospital, Arnhem, the Netherlands (J.H.).; Department of Neurology, Radboud University Medical Center, Nijmegen, the Netherlands (F.S.).; Department of Neurology, Zuyderland Medical Center, Heerlen, the Netherlands (T.A.H.C.M.L.S.).; Department of Neurology (Y.B.W.E.M.R.), Amsterdam University Medical Centers, the Netherlands.; Department of Radiology and Nuclear Medicine (C.B.L.M.M.), Amsterdam University Medical Centers, the Netherlands.; Department of Neurology, University of Michigan, Ann Arbor (J.F.B.).; Department of Radiology and Nuclear Medicine, Leiden University Medical Center, the Netherlands (A.C.G.M.v.E.).

**Keywords:** blood pressure, cerebral hemorrhage, guideline, ischemic stroke, reperfusion

## Abstract

**Background and Purpose:**

Optimal blood pressure (BP) management in the acute phase of ischemic stroke remains an unresolved issue. It is uncertain whether guidelines for BP management during and after intravenous alteplase can be extrapolated to endovascular treatment (EVT) for stroke due to large artery occlusion in the anterior circulation. We evaluated the associations between systolic BP (SBP) in the first 6 hours following EVT and functional outcome as well as symptomatic intracranial hemorrhage.

**Methods:**

Patients of 8 MR CLEAN (Multicenter Randomized Controlled Trial of Endovascular Treatment for Acute Ischemic Stroke in the Netherlands) Registry centers, with available data on SBP in the 6 hours following EVT, were analyzed. We evaluated maximum, minimum, and mean SBP. Study outcomes were functional outcome (modified Rankin Scale) at 90 days and symptomatic intracranial hemorrhage. We used multivariable ordinal and binary regression analysis to adjust for important prognostic factors and studied possible effect modification by successful reperfusion.

**Results:**

Post-EVT SBP data were available for 1161/1796 patients. Higher maximum SBP (per 10 mm Hg increments) was associated with worse functional outcome (adjusted common odds ratio, 0.93 [95% CI, 0.88–0.98]) and a higher rate of symptomatic intracranial hemorrhage (adjusted odds ratio, 1.17 [95% CI, 1.02–1.36]). The association between minimum SBP and functional outcome was nonlinear with an inflection point at 124 mm Hg. Minimum SBP lower and higher than the inflection point were associated with worse functional outcomes (adjusted common odds ratio, 0.85 per 10 mm Hg decrements [95% CI, 0.76–0.95] and adjusted common odds ratio, 0.81 per 10 mm Hg increments [95% CI, 0.71–0.92]). No association between mean SBP and functional outcome was observed. Successful reperfusion did not modify the relation of SBP with any of the outcomes.

**Conclusions:**

Maximum SBP in the first 6 hours following EVT is positively associated with worse functional outcome and an increased risk of symptomatic intracranial hemorrhage. Both lower and higher minimum SBP are associated with worse outcomes. A randomized trial to evaluate whether modifying post-intervention SBP results in better outcomes after EVT for ischemic stroke seems justified.

## Abstract 2

Supplemental Digital Content is available in the text.

In the first 24 hours after stroke, BP is often increased, even after endovascular treatment (EVT), and it takes a few days to return to baseline levels.^1,2^ It has been demonstrated that admission BP is strongly associated with functional outcome after EVT.^3–5^ Since BP is an important factor affecting cerebral perfusion, it is likely that BP within the first hours following EVT has an impact on infarct size and thereby functional outcome.^6,7^ Two observational studies found an association between systolic BP (SBP) peaks in the 24 hours following stroke and increased risks of symptomatic intracranial hemorrhage (sICH) and functional dependency.^2,8^ However, these studies did not relate timing of BP measurement to the occurrence of sICH, so reverse causality could be present and the target BP level in the first few hours after EVT remains unclear. Yet, if BP is causally related to outcome, modification using medication might be a feasible strategy to improve functional outcomes. We aimed to evaluate the associations of SBP in the first 6 hours following EVT with functional outcome and the occurrence of sICH.

## Methods

### Study Protocol and Data Availability

We used data from the MR CLEAN (Multicenter Randomized Controlled Trial of Endovascular Treatment for Acute Ischemic Stroke in the Netherlands) Registry, a prospective, multicenter, observational cohort, including all consecutive patients treated with EVT for acute ischemic stroke in the Netherlands between March 2014 and 2017. Detailed information on the description of variables and the methods of MR CLEAN Registry have been reported previously.^9^ Data cannot be made available, as no patient approval has been obtained for sharing coded data. However, R syntax and output files of the analyses will be made available on request.

### Study Population

Patients were eligible for inclusion for this analysis if they had been treated in an MR CLEAN Registry center that was able to provide BP data of the first 6 hours after EVT. Individual patients were included if they were 18 years or older; had a proximal intracranial occlusion in the anterior circulation (intracranial carotid artery/intracranial carotid artery terminus, middle cerebral artery [M1/M2], and anterior cerebral artery [A1/A2]) confirmed on computed tomography angiography; in whom groin puncture was possible within 6.5 hours after symptom onset; and had at least one available BP value within the first 6 hours following EVT.

### BP Measures

We collected SBP values recorded between the end of the EVT procedure (defined as time of reperfusion or last contrast bolus) and 24 hours after EVT or until discharge from the intervention center. To limit the risk of confounding by indication based on missing BP data due to early transfer of patients in good condition, we restricted our primary analysis to the first 6 hours following EVT. The predefined BP measures of interest included (1) maximum SBP (reflecting peak in BP course), (2) minimum SBP (reflecting drops in BP), and (3) mean SBP. If >1 SBP measurement was available, maximum and minimum SBP were calculated based on the average of the 2 highest or lowest SBP values in the 6 hours following EVT, to limit the risk of measurement error. When only one SBP value was available, there was no difference between maximum, minimum and mean SBP. Additionally, we performed a sensitivity analysis to evaluate the association between the predefined BP measures in the first 24 hours following EVT and outcomes. Since the majority of sICH and extracranial hemorrhage occurs within 24 hours following EVT, we did not evaluate the association between BP and these outcomes to avoid reverse causality. Details on BP protocols of the included centers are described in Table I in the Data Supplement.

### Outcome Measures

The primary outcome measure was functional outcome according to the modified Rankin Scale, which is a 7-point scale ranging from 0 no symptoms to 6 death, assessed at 90 days after EVT.^10^ Secondary outcome measures included functional independence (modified Rankin Scale score ≤2), mortality within 90 days after EVT, National Institutes of Health Stroke Scale score indicating neurological deficit at 24 to 48 hours after EVT, extracranial hemorrhage (requiring surgery or blood transfusion), and new ischemic stroke (new neurological deficit confirmed with imaging) within 90 days from stroke onset. Furthermore, any occurrence of sICH (neurological deterioration of ≥4 points on the National Institutes of Health Stroke Scale and a compatible hemorrhage on noncontrast computed tomography assessed by an independent core laboratory according to the Heidelberg criteria) was included as a secondary outcome measure.^11,12^

### Statistical Analysis

Baseline characteristics of the study population are tabulated by 3 subgroups according to maximum SBP tertiles. Continuous variables are expressed as means (SD) or medians (interquartile ranges), where applicable. Categorical variables are expressed as numbers of patients and percentages.

We evaluated the linearity of the associations between the postprocedural SBP parameters and outcomes by comparing model fit of a regression model with a linear SBP term to a regression model with a SBP term with a restricted cubic spline transformation with 3 knots. We performed multivariable ordinal logistic regression, binary logistic regression or linear regression analyses, as appropriate with adjustment for the following potential confounders: age, sex, National Institutes of Health Stroke Scale score on admission, prestroke modified Rankin Scale score, medical history of hypertension, stroke, diabetes, atrial fibrillation, myocardial infarction, treatment with intravenous thrombolysis, SBP on admission, location of occlusion, Alberta Stroke Program Early CT Score on noncontrast computed tomography,^13^ collateral score on computed tomography angiography according to a 4-point scale (0=absent collaterals [0% filling of the vascular territory downstream of the occlusion], 1=poor collaterals [>0% and ≤50% filling], 2=moderate collaterals [>50% and <100% filling], and 3=excellent collaterals [100% filling]),^14^ the use of general anesthesia during EVT, time from stroke onset to reperfusion or last contrast bolus, extended Thrombolysis in Cerebral Infarction score at the end of the EVT procedure,^15^ number of BP measurements in the 6 hours following EVT, and intervention center. For the outcome sICH, we aimed to reduce the possibility that results were hampered by reverse causality (ie, BP measurements collected during or after occurrence of sICH) by excluding patients in whom sICH occurred within 6 hours following EVT. The associations of BP parameters with outcomes were presented per 10 mm Hg change in BP.

We assessed whether the relation between postprocedural BP and outcomes was modified by the extent of reperfusion. We fitted a similar multivariable regression model as described above including an interaction term for SBP parameter*successful reperfusion, a dichotomized term for extent of reperfusion (unsuccessful, extended Thrombolysis in Cerebral Infarction score <2B versus successful, extended Thrombolysis in Cerebral Infarction score ≥2B).^15^ For all regression analyses, missing data were imputed using multiple imputations by chained equations based on relevant covariates and outcomes.^16^ All analyses were performed using R software (Version 3.6.1, R foundation for Statistical Computing, Vienna, Austria) with the packages: *tableone*, *mice*, *Hmisc*, *ggplot*, and *rms*.

### Medical Ethics Committee Statement

The medical ethics committee of the Erasmus University MC, Rotterdam, the Netherlands, evaluated the study protocol of the MR CLEAN Registry and granted permission to perform the study as a registry (MEC-2014-235).

## Results

### Study Population

**Figure 1.**
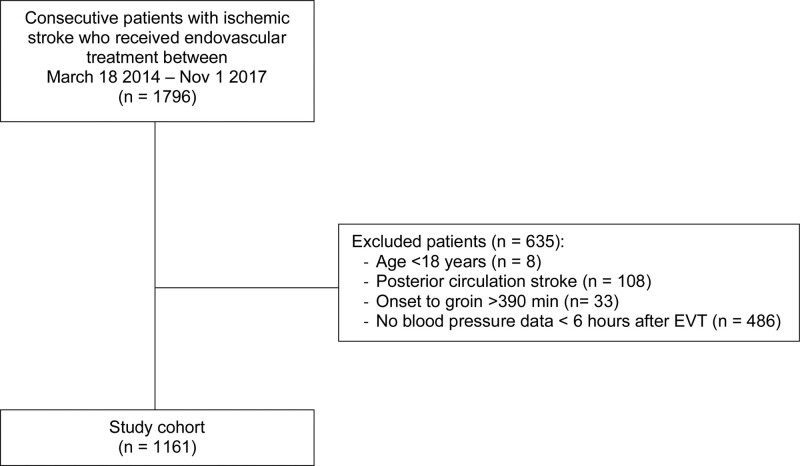
**Flowchart of patient inclusion.** EVT indicates endovascular treatment.

Of 1796 patients treated with EVT during the study period in the 8 participating centers, 1161 (65%) were included in the current analysis (Figure 1). The median available number of SBP measurements in the first 6 hours following EVT was 7 (interquartile range, 4–11). For 86/1161 patients only one SBP value in the first 6 hours was available. The mean SBP in the first 6 hours following EVT was 150 mm Hg (SD 25). Baseline characteristics of the study population are shown according to maximum SBP tertiles (Table 1). Patients with a higher maximum SBP in the first 6 hours following EVT were on average older and were more likely to have a history of atrial fibrillation, diabetes, hypertension, distal occlusion, and poorer collateral scores.

**Table 1.**
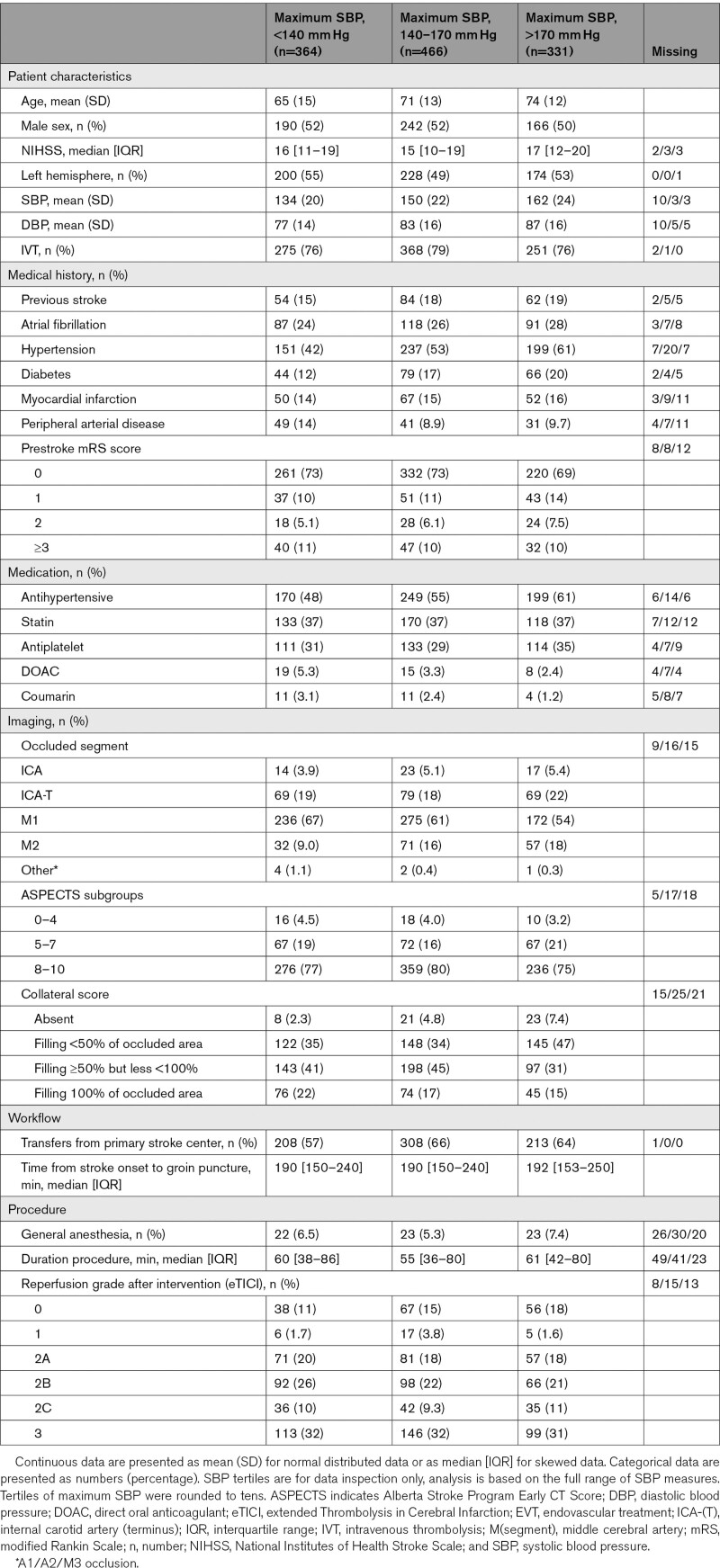
Baseline Characteristics of All Patients Shown According to Tertiles of Maximum SBP During First 6 Hours Following EVT

### Association of Maximum SBP With Outcomes

**Figure 2.**
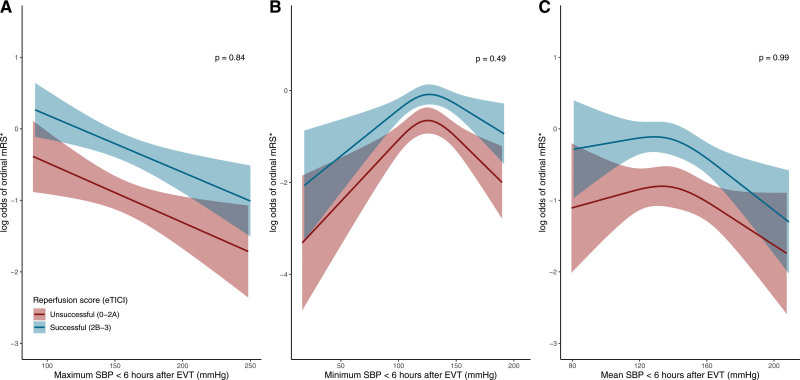
**Relationship of systolic blood pressure (SBP) and shift toward better functional outcome.** The models are fitted with a linear function for maximum SBP and restricted cubic spline function with 3 knots for minimum SBP and mean SBP parameters. All models include the following variables: age, National Institutes of Health Stroke Scale (NIHSS) at baseline, Alberta Stroke Program Early CT Score (ASPECTS) at baseline, history of hypertension, time between stroke onset to reperfusion, and an interaction term for SBP parameter*reperfusion grade. The graphs depict the log odds for a shift towards better modified Rankin Scale (mRS) score (*ordinal mRS) with 95% CI, for each level of maximum SBP (**A**), minimum SBP (**B**), and mean SBP (**C**) in the first 6 h following endovascular treatment (EVT) for successful and unsuccessful reperfusion, with corresponding *P* value for interaction. The ranges of the *x* axes correspond to the lowest and highest SBP value in the data. eTICI indicates extended Thrombolysis in Cerebral Infarction.

The association between maximum SBP and functional outcome at 90 days (shift towards better modified Rankin Scale score) was linear (Figure 2A, likelihood ratio test *P*=0.14 for maximum SBP). Patients with higher maximum SBP in the 6 hours following EVT were more likely to have worse functional outcomes compared with patients with lower maximum SBP (adjusted common odds ratio [OR], 0.93 per 10 mm Hg [95% CI, 0.88–0.98], Table 2). Higher maximum SBP was associated with a larger neurological deficit (measured with the National Institutes of Health Stroke Scale) at 24 to 48 hours after EVT (aβ 0.31 [95% CI, 0.14–0.49]), increased risk of sICH (adjusted OR, 1.17 [95% CI, 1.02–1.36]), but not with an increased risk of death (adjusted OR, 1.02 [95% CI, 0.95–1.08], Table 2). In the sensitivity analysis of SBP measures during the first 24 hours, we observed a similar association between higher maximum SBP and worse functional outcome (adjusted common OR, 0.90 per 10 mm Hg [95% CI, 0.85–0.94], Table II in the Data Supplement).

**Table 2.**
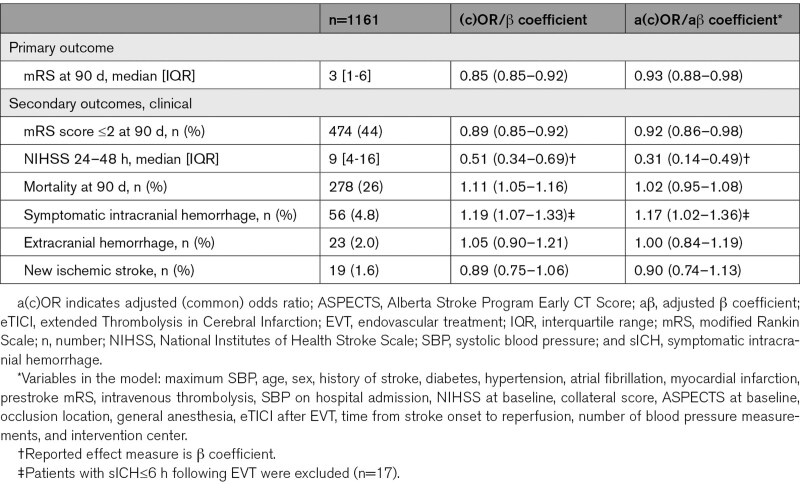
Associations Between Continuous Maximum SBP Within First 6 Hours Following EVT and Outcomes Shown per 10 mm Hg Increment in SBP

### Association of Minimum SBP With Outcomes

The association between minimum SBP and functional outcome was nonlinear (Figure 2B) based on multivariable model fit comparing a linear SBP term to a model allowing 3 knots for SBP (likelihood ratio test *P*<0.01 for minimum SBP). Due to the nonlinearity of this association, we obtained effect estimates for lower minimum and higher minimum SBP separately (inflection point at around 124 mm Hg). Minimum SBP below 124 mm Hg and minimum SBP above 124 mm Hg were both associated with worse functional outcome (adjusted common OR per 10 mm Hg decrement 0.85 [95% CI, 0.76–0.95] for minimum SBP <124 mm Hg and 0.81 per 10 mm Hg increment [95% CI, 0.71–0.92] for minimum SBP ≥124 mm Hg). Also, minimum SBP lower than 124 mm Hg and minimum SBP higher than 124 mm Hg were associated with higher mortality rates and a more frequent occurrence of extracranial hemorrhage. Minimum SBP higher than 124 mm Hg was associated with more neurological deficit at 24 to 48 hours, which was not observed for lower minimum SBP (Table III in the Data Supplement).

### Association of Mean SBP With Outcomes

The associations between mean SBP and functional outcome were also nonlinear (Figure 2C) based on multivariable model fit comparing a linear SBP term to a model allowing 3 knots for SBP (likelihood ratio test *P*<0.01 for mean SBP). Therefore, we obtained effect estimates for lower mean SBP and higher mean SBP separately (inflection point at around 138 mm Hg). Mean SBP below 138 mm Hg was associated with higher likelihood of extracranial hemorrhage (adjusted OR, 1.66 per 10 mm Hg decrement [95% CI, 1.07–2.51]). We did not observe an association between mean SBP higher than 138 mm Hg and any of the outcomes (Table IV in the Data Supplement). The distribution of outcomes according to maximum, minimum and mean SBP tertiles is shown in Figure I and Table V in the Data Supplement.

We did not find an interaction between extend of reperfusion and the relation of SBP with functional outcome (*P* values for interaction: maximum SBP=0.84; minimum SBP=0.49 and mean SBP=0.99, Figure 2) or any of the secondary outcomes (Figure II in the Data Supplement). We observed a decline in maximum SBP from baseline during the 6 hours following EVT for both reperfusion categories, with higher maximum SBPs among patients with unsuccessful reperfusion at the end of EVT procedure compared with patients with successful reperfusion (Figure III in the Data Supplement).

## Discussion

Increased maximum SBP in the first 6 hours following EVT was associated with worse functional outcome, a greater risk of sICH and more severe early neurological deficits. Minimum SBP lower and higher than the inflection point of 124 mm Hg were associated with worse functional outcome. A mean SBP lower than 138 mm Hg was associated with an increased risk of extracranial hemorrhage. None of the associations between BP and outcomes were modified by successful reperfusion at the end of the EVT procedure.

Our results are in line with previous studies reporting that higher maximum SBPs in the 24 hours following EVT are associated with worse clinical outcomes.^2,8,17–19^ The explanation for the worse outcome observed in patients with higher maximum SBP is likely to be multifactorial, including disruption of the blood-brain barrier, hemorrhagic transformation, elevated serum catecholamine levels, and larger infarcts.^20^ The association between higher BP and worse outcomes following EVT has been observed up to 3 days after treatment, stressing the importance of patient monitoring and support following EVT.^21^ In contrast with our findings, no association between maximum SBP after EVT and risk of sICH was observed in a subgroup analysis of a recent meta-analysis including 791 patients.^19^

We observed a nonlinear association between minimum SBP and functional outcome, with an inflection point at 124 mm Hg during the first 6 hours following EVT. Previous studies evaluating minimum SBP did not find an association with functional outcome. However, these studies were small, no test for nonlinearity was performed, and functional outcome was assessed dichotomously.^18,22^ Only one other study reported that an increase in minimum SBP was associated with an increased likelihood of functional independence.^17^ Low SBP in the (sub)acute phase of ischemic stroke might be associated with impaired cerebral perfusion, infarct expansion, or complications like impending sepsis.^22,23^

We observed a small decrease of maximum SBP following EVT in patients with successful compared to unsuccessful reperfusion, similar to previous findings.^1^ It has been hypothesized that optimal BP regime varies with the reperfusion status (ie, successful or unsuccessful). For example, higher SBP might be associated with hemorrhagic transformation given complete reperfusion.^24,25^ However, maintaining hypertension might be of benefit in patients with unsuccessful reperfusion to optimize collateral blood flow and maintain cerebral perfusion pressure.^7,17,26^ Several studies reported modification of the effect of BP on outcome by reperfusion status.^18,22^ However, in our large study cohort, we did not observe different associations between SBP and functional outcome for patients with successful and unsuccessful reperfusion, which was also observed by another cohort study.^2^ This might partially be explained by the fact that high SBP is a marker of tissue damage rather than reperfusion success. Therefore, successful reperfusion should probably be regarded as a confounder of the association between BP and outcome and not only as an effect modifier.

Given the clear association between BP and outcome after EVT, the lack of evidence on optimal BP management, the variation in hemodynamic management among EVT centers, and the possibility of a modifiable effect of BP on outcome, a clinical trial seems justified.^27^ Currently, the BEST-II trial (Blood Pressure After Endovascular Stroke Therapy-II; URL: https://www.clinicaltrials.gov; Unique identifier: NCT04116112) aims to evaluate the safety of lower SBP in patients treated with EVT in whom successful reperfusion is achieved. In this trial, patients will be randomly assigned to one of the following SBP targets: ≤180, <160, and <140 mm Hg. Intravenous antihypertensive treatment will be started after reperfusion to maintain SBP below the assigned target for 24 hours.^28^

Furthermore, the BP-TARGET trial (Blood Pressure Target in Acute Stroke to Reduce Hemorrhage After Endovascular Therapy; URL: https://www.clinicaltrials.gov; Unique identifier: NCT03160677) aims to determine whether strict SBP control (intervention arm: SBP between 110 and 129 mm Hg) versus standard SBP control (control arm: SBP between 130 and 185 mm Hg) during 24 hours following EVT in patients with successful reperfusion will reduce the risk of any intracranial hemorrhage.^29,30^ Besides, the ongoing MR ASAP trial (Multicentre Randomised Trial of Acute Stroke Treatment in the Ambulance With Nitroglycerin Patch) aims to assess the effect of transdermal glyceryl trinitrate started within 3 hours of symptom onset in the prehospital setting on functional outcome in patients with ischemic stroke or intracerebral hemorrhage. This intervention is suggested to improve outcome after stroke by an increase in the intracranial collateral flow and a reduction of the BP.^31^ Although these further studies on hemodynamic management in stroke patients are warranted, one of the major challenges of hemodynamic management remains to extrapolate population-based data to determine the target BP for an individual stroke patient.

### Limitations

Our study has several limitations. First, due to the retrospective observational design, results could have been confounded by variables not adjusted for in the analyses, so residual confounding might be present. Second, our observed associations do not prove causality between SBP and outcome measures. SBP could have been measured during the asymptomatic phase preceding sICH. Hence, definitive inferences on effects of SBP treatment are not possible. Furthermore, as we did not have data on individual SBP targets or information on administration of either a vasopressor or an antihypertensive agent after EVT, we do not know how well SBP was managed. Besides, as data on follow-up infarct volumes were not available systematically, we could not evaluate if patients with higher SBP were more likely to have larger infarcts.

### Conclusions

Patients with higher maximum SBP in the 6 hours following EVT are more likely to have worse functional outcome or sICH compared with patients with lower maximum SBP. Lower as well as higher minimum SBP are associated with worse functional outcome. Randomized trials are needed to evaluate whether modifying SBP post-EVT improves outcome.

## Acknowledgments

We thank the MR CLEAN (Multicenter Randomized Controlled Trial of Endovascular Treatment for Acute Ischemic Stroke in the Netherlands) Registry investigators. Drs Samuels, van de Graaf, and Dippel performed the study concept, statistical analysis, interpretation of the results, and drafting of the article. C.A.L. van den Berg, K. Bala, Dr van der Steen, Dr Witvoet, Dr den Hertog, Dr Schonewille, Dr Hofmeijer, Dr Schreuder, Dr Schreuder, Dr Roos, and Dr Majoie performed data aquisition and critical revision of the article. Drs van der Worp, Boiten, van Es, van Doormaal, Roozenbeek, Uniken Venema, Lingsma, Burke, van der Lugt, and Dippel performed the critical revision of the article.

## Sources of Funding

The MR CLEAN (Multicenter Randomized Controlled Trial of Endovascular Treatment for Acute Ischemic Stroke in the Netherlands) Registry was partially funded by unrestricted grants from Toegepast Wetenschappelijk Instituut voor Neuromodulatie, Twente University (TWIN), Erasmus MC University Medical Center, Maastricht University Medical Center, and Amsterdam UMC. The study was additionally funded by the European Union’s Horizon 2020 research and innovation program under grant agreement no. 777072 (INSIST [In-Silico Trials for Treatment of Acute Ischemic Stroke]). The funding sources had no role in study design, patient enrolment, data collection, analysis, writing of the article, approval of the article, and decision to submit the article for publication.

## Disclosures

Dr Dippel reports funding from the Dutch Heart Foundation, Brain Foundation Netherlands, The Netherlands Organisation for Health Research and Development, Health Holland Top Sector Life Sciences and Health, and unrestricted grants from Penumbra Inc, Stryker European Operations BV, Medtronic, Thrombolytic Science, LLC and Cerenovus for research, all paid to the institution. Dr van der Lugt reports funding from Dutch Heart Foundation, Dutch Brain Foundation, Stryker, Angiocare BV, Medtronic/Covidien/EV3, MEDAC Gmbh/LAMEPRO, Penumbra, Cerenovus, Thrombolytic Science LLC, and Top Medical Concentric, all paid to institution. Dr van Doormaal reports funding from Stryker, paid to institution. Dr Burke reports grants from the National Institutes of Health (NIH). Dr Majoie reports funding from CVON/Dutch Heart Foundation, Stryker, Health Evaluation Netherlands all paid to institution and is shareholder of Nico.lab, a company that focuses on the use of artificial intelligence for medical imaging analysis. Dr Roos reports funding from CVON/Dutch Heart Foundation, Stryker, Health Evaluation Netherlands all paid to institution and reports being shareholder of Nico.lab, a company that focuses on the use of artificial intelligence for medical imaging analysis. Dr Schreuder reports grants from the Dutch Heart Foundation. Dr van der Worp reports funding from Bayer, Boehringer Ingelheim, and LivaNova for consultation, grants from Stryker and Dutch Heart Foundation, all paid to institution. The other authors report no conflicts.

## Supplemental Materials

Online Tables I–V

Online Figures I–III

List of group authors

## Supplementary Material


