# Supplementary material for: Blood Pressure in the First 6 Hours Following Endovascular Treatment for Ischemic Stroke Is Associated With Outcome
Source: Stroke. 2021 Sep 20;52(11):3514–22. doi: 10.1161/STROKEAHA.120.033657 (PMC8547588; doi:10.1161/STROKEAHA.120.033657)
Supplement: Supplementary file 1 [file str-52-3514-s001.pdf]

SUPPLEMENTAL MATERIAL

BLOOD PRESSURE IN THE FIRST 6 HOURS FOLLOWING ENDOVASCULAR TREATMENT FOR ISCHEMIC STROKE IS ASSOCIATED WITH OUTCOME

Table I. Overview of protocols for blood pressure management in the 8 included centers

Center	Periprocedural blood pressure targets* (SBP/DBP)	Frequency of blood pressure measurements during 24 hours following EVT†	First and second preferred agent to treat arterial hypertension during first 24 hours after EVT‡
1	<185/110 mmHg	0-6: hourly<br>6-24 hours: 2-hourly<br>NB IVT first hour every 15 minutes	Labetalol, nicardipine
2	<185/110 mmHg	0-24: hourly	Labetalol
3	<185/110 mmHg	0-2 hours: 15 minutes<br>2-10 hours: 2-hourly<br>10-24 hours: 4-hourly	Labetalol, nicardipine
4	<185/110 mmHg	0-2 hours: 15 minutes<br>2-6 hours: 30 minutes<br>6-24 hours: hourly	Labetalol
5	<185/110 mmHg	0-2 hours: 15 minutes<br>2-6 hours: 30 minutes<br>6-24 hours: hourly	Labetalol, nicardipine
6	<185/110 mmHg	0-2 hours: 15 minutes<br>2-8 hours: 30 minutes<br>8-24 hours: hourly	Nifedipine, labetalol
7	<185/110 mmHg	0-2 hours: 15 minutes<br>2-6 hours: 30 minutes<br>6-24 hours: hourly	Labetalol, perindopril/nifedipine
8	<185/110 mmHg	0-2 hours: 15 minutes<br>2-8 hours: 30 minutes<br>8-24 hours: hourly	Labetalol, clonidine

Abbreviations: DBP, diastolic blood pressure; EVT, endovascular treatment; SBP, systolic blood pressure.
*Non-invasive blood pressure monitoring. † Preprocedural, procedural, and up to 24 hours post-procedural SBP targets. ‡Might be different from the preferred agents for lowering blood pressure prior to intravenous alteplase administration or EVT.

Table II. Associations between continuous maximum SBP within first 24 hours following EVT and outcomes shown per 10mmHg increment in SBP (full cohort, n= 1161).		
	(c)OR / β-coefficient, (95% CI)	a(c)OR / aβ-coefficient, (95% CI)*
Primary outcome		
mRS at 90 days	0.85 (0.82 to 0.89)	0.90 (0.85 to 0.94)
Secondary outcomes		
mRS ≤2 at 90 days	0.85 (0.82 to 0.90)	0.89 (0.83 to 0.95)
NIHSS 24–48 hours †	0.77 (0.58 to 0.95)	0.51 (0.31 to 0.70)
Mortality at 90 days	1.15 (1.10 to 1.21)	1.06 (0.99 to 1.14)
New ischemic stroke	0.89 (0.74 to 1.06)	0.92 (0.73 to 1.18)

Abbreviations: aβ, adjusted beta-coefficient; a(c)OR, adjusted (common) odds ratio; CI, confidence interval; EVT, endovascular treatment; IQR, interquartile range; mRS, modified Rankin Scale; NIHSS, National Institutes of Health Stroke Scale; SD, standard deviation; SBP, systolic blood pressure. *Variables in the model: maximum SBP, age, sex, history of stroke, diabetes mellitus, hypertension, atrial fibrillation, myocardial infarction, pre-stroke mRS, intravenous thrombolysis, SBP on hospital admission, NIHSS at baseline, collateral score, ASPECTS at baseline, occlusion location, general anesthesia, eTICI score, time between stroke onset to reperfusion, number of blood pressure measurements, and intervention center. †Reported effect measure is β-coefficient.

Table III. Associations between continuous minimum SBP within first 6 hours following EVT and outcomes.				
	Minimum SBP < 124mmHg per 10mmHg decrement in SBP		Minimum SBP ≥ 124mmHg per 10mmHg increment in SBP	
	(c)OR/ β-coefficient, (95% CI)*	a(c)OR/ aβ-coefficient, (95% CI)*†	(c)OR/β-coefficient, (95% CI)*	a(c)OR/aβ-coefficient, (95% CI)*†
Primary outcome				
mRS at 90 days	0.86 (0.78 to 0.95)	0.85 (0.76 to 0.95)	0.76 (0.67 to 0.86)	0.81 (0.71 to 0.92)
Secondary outcomes				
mRS ≤2 at 90 days	0.88 (0.79 to 0.99)	0.90 (0.78 to 1.04)	0.79 (0.67 to 0.90)	0.89 (0.73 to 1.04)
NIHSS 24–48 hours ‡	0.74 (-0.26 to 1.23)	0.41 (-0.02 to 0.84)	1.44 (0.83 to 2.05)	0.76 (0.23 to 1.28)
Mortality at 90 days	1.20 (1.06 to 1.34)	1.22 (1.04 to 1.40)	1.33 (1.15 to 1.54)	1.23 (1.01 to 1.45)
Symptomatic intracranial hemorrhage §	1.11 (0.80 to 1.43)	1.17 (0.83 to 1.57)	1.32 (0.93 to 1.79)	1.34 (0.91 to 1.90)
Extracranial hemorrhage	1.61 (1.26 to 2.05)	1.71 (1.27 to 2.30)	1.78 (1.24 to 2.50)	1.80 (1.21 to 2.67)
New ischemic stroke	1.30 (0.92 to 1.71)	1.38 (0.91 to 1.93)	1.30 (0.79 to 1.93)	1.35 (0.78 to 2.19)

Abbreviations: aβ, adjusted beta-coefficient; a(c)OR, adjusted (common) odds ratio; CI, confidence interval; EVT, endovascular treatment; mRS, modified Rankin Scale; NIHSS, National Institutes of Health Stroke Scale; SBP, systolic blood pressure. *regression models include a restricted cubic spline function with 3 knots for the continuous minimum SBP term. †Variables in the model: minimum SBP, age, sex, history of stroke, diabetes mellitus, hypertension, atrial fibrillation, myocardial infarction, pre-stroke mRS, intravenous thrombolysis, SBP on hospital admission, NIHSS at baseline, collateral score, ASPECTS at baseline, occlusion location, general anesthesia, eTICI after EVT, time from stroke onset to reperfusion, number of blood pressure measurements, and intervention center. ‡Reported effect measure is β-coefficient. §Patients with sICH ≤6 hours following EVT were excluded (n=17).

Table IV. Associations between continuous mean SBP within first 6 hours following EVT and outcomes.				
	Mean SBP< 138mmHg per 10mmHg decrement		Mean SBP ≥ 138mmHg per 10mmHg increment	
	(c)OR/ β-coefficient, (95% CI)*	a(c)OR/ aβ-coefficient, (95% CI)*†	(c)OR/β-coefficient, (95% CI)*	a(c)OR/aβ-coefficient, (95% CI)*†
Primary outcome				
mRS at 90 days	1.01 (0.90 to 1.13)	0.97 (0.86 to 1.10)	0.83 (0.72 to 0.96)	0.88 (0.76 to 1.03)
Secondary outcomes				
mRS ≤2 at 90 days	1.05 (0.92 to 1.18)	1.06 (0.88 to 1.23)	0.90 (0.74 to 1.03)	1.01 (0.78 to 1.19)
NIHSS 24–48 hours ‡	0 (-0.54 to 0.55)	-0.07 (-0.56 to 0.41)	0.86 (0.16 to 1.56)	0.40 (-0.20 to 0.99)
Mortality at 90 days	1.04 (0.90 to 1.21)	1.13 (0.95 to 1.36)	1.26 (1.06 to 1.51)	1.20 (0.97 to 1.49)
Symptomatic intracranial hemorrhage §	0.94 (0.61 to 1.35)	1.06 (0.66 to 1.56)	1.18 (0.75 to 1.77)	1.29 (0.79 to 1.99)
Extracranial hemorrhage	1.50 (1.02 to 2.10)	1.66 (1.07 to 2.51)	1.61 (0.98 to 2.52)	1.55 (0.90 to 2.58)
New ischemic stroke	1.49 (0.99 to 2.16)	1.66 (0.98 to 2.52)	1.53 (0.86 to 2.52)	1.73 (0.88 to 3.11)

Abbreviations: aβ, adjusted beta-coefficient; a(c)OR, adjusted (common) odds ratio; CI, confidence interval; EVT, endovascular treatment; mRS, modified Rankin Scale; NIHSS, National Institutes of Health Stroke Scale; SBP, systolic blood pressure. *regression models include a restricted cubic spline function with 3 knots for the continuous mean SBP term. †Variables in the model: mean SBP, age, sex, history of stroke, diabetes mellitus, hypertension, atrial fibrillation, myocardial infarction, pre-stroke mRS, intravenous thrombolysis, SBP on hospital admission, NIHSS at baseline, collateral score, ASPECTS at baseline, occlusion location, general anesthesia, eTICI after EVT, time from stroke onset to reperfusion, number of blood pressure measurements, and intervention center. ‡Reported effect measure is β-coefficient. §Patients with symptomatic intracranial hemorrhage ≤6 hours following EVT were excluded (n=17).

Table V. Outcomes shown according to tertiles of SBP during first 6 hours following EVT.

	Maximum SBP < 140 mmHg (n = 365)	*Maximum SBP 140 - 170 mmHg (n = 461)*	*Maximum SBP >170 mmHg (n = 335)*	*Missing*
Outcome measures				
mRS ≤2 at 90 days, n (%)	184 (54)	189 (45)	101 (32)	25/37/22
NIHSS 24–48 hours, median [IQR]	8 [3 to 14]	10 [4 to 16]	12 [5 to 18]	18/23/21
Mortality at 90 days, n (%)	68 (20)	101 (24)	109 (35)	25/37/22
Symptomatic intracranial hemorrhage, n (%)	12 (3.3)	13 (2.8)	31 (9.3)	
Extracranial hemorrhage, n (%)	6 (1.6)	11 (2.4)	6 (1.8)	
New ischemic stroke, n (%)	7 (1.9)	8 (1.7)	4 (1.2)	
	Minimum SBP < 110 mmHg (n = 322)	*Minimum SBP 110 - 130 mmHg (n = 413)*	*Minimum SBP >130 mmHg (n = 426)*	*Missing*
mRS ≤2 at 90 days, n (%)	126 (43)	183 (48)	165 (41)	32/28/24
NIHSS 24–48 hours, median [IQR]	10 [4 to 16]	9 [3 to 15]	11 [4 to 17]	14/18/30
Mortality at 90 days, n (%)	81 (28)	84 (22)	113 (28)	32/28/24
Symptomatic intracranial hemorrhage, n (%)	16 (5)	10 (2.4)	30 (7.0)	
Extracranial hemorrhage, n (%)	13 (4.0)	3 (0.7)	7 (1.6)	
New ischemic stroke, n (%)	8 (2.5)	4 (1.0)	7 (1.6)	
	Mean SBP < 130 mmHg (n = 423)	*Mean SBP 130 - 150 mmHg (n = 370)*	*Mean SBP >150 mmHg (n = 368)*	*Missing*
mRS ≤2 at 90 days, n (%)	200 (52)	151 (44)	123 (36)	35/25/24
NIHSS 24–48 hours, median [IQR]	8 [3 to 15]	9 [4 to 16]	12 [5 to 18]	23/15/24
Mortality at 90 days, n (%)	81 (21)	80 (23)	117 (34)	35/25/24
Symptomatic intracranial hemorrhage, n (%)	11 (2.6)	13 (3.5)	32 (8.7)	
Extracranial hemorrhage, n (%)	13 (3.1)	3 (0.8)	7 (1.9)	
New ischemic stroke, n (%)	8 (1.9)	7 (1.9)	4 (1.1)	

Abbreviations: IQR, interquartile range; min, minutes; mRS, modified Rankin Scale; n, number; NIHSS, National Institutes of Health Stroke Scale; SD, standard deviation; SBP, systolic blood pressure

Captions: *Tertiles of SBP were rounded to tens. Continuous data are presented as mean (SD) for normal distributed data or as median [IQR] for skewed data. Categorical data are presented as numbers (percentage).

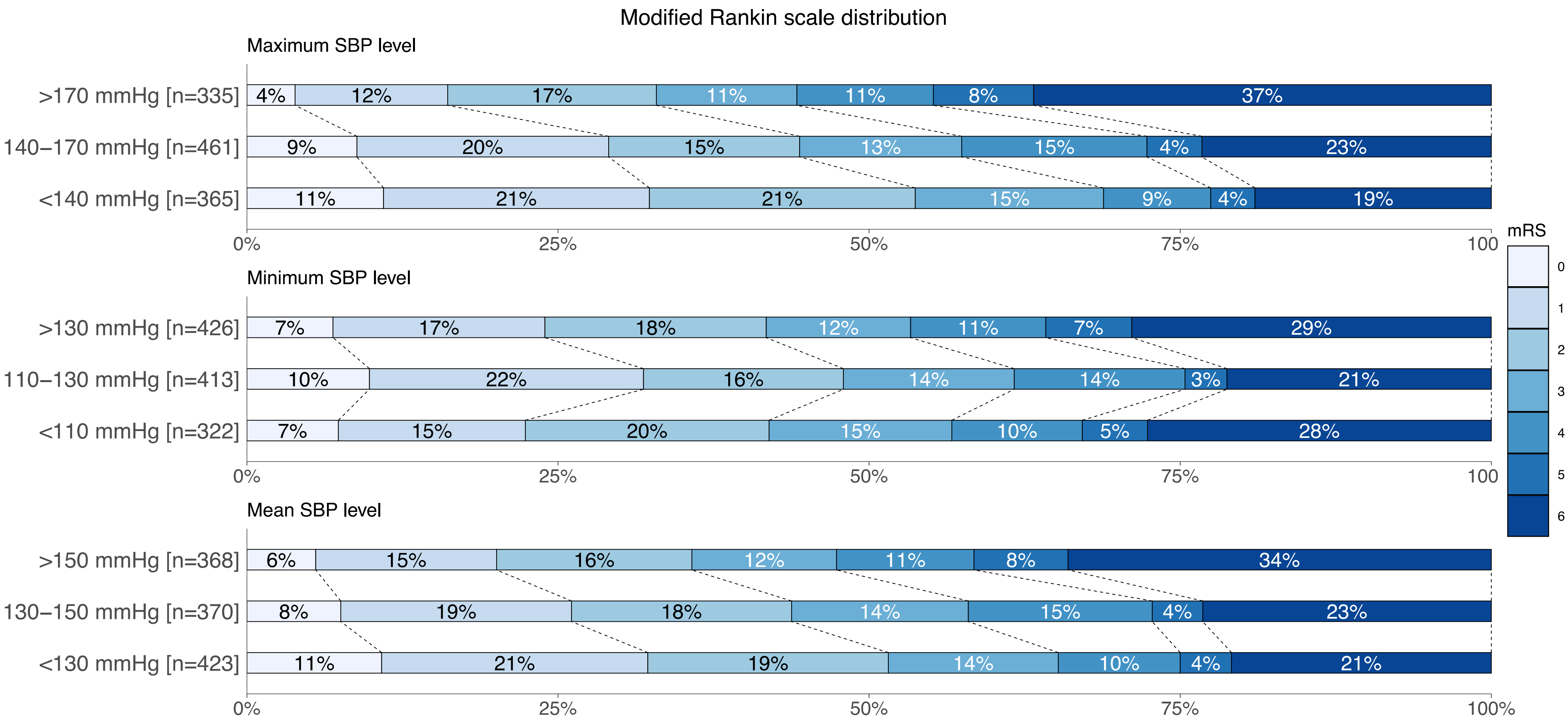

Tertiles of SBP parameters were rounded to tens. No analyses were performed according to SBP tertiles, distribution of mRS for illustration only.

Figure I. Distribution of modified Rankin scale according to tertiles of maximum, minimum and mean SBP during the first 6 hours following EVT. SBP tertiles are used for data inspection only, analysis is based on the fulle range of SBP measures.
Abbreviations: EVT, endovascular treatment; mRS, modified Rankin Scale; n, number; SBP, systolic blood pressure.

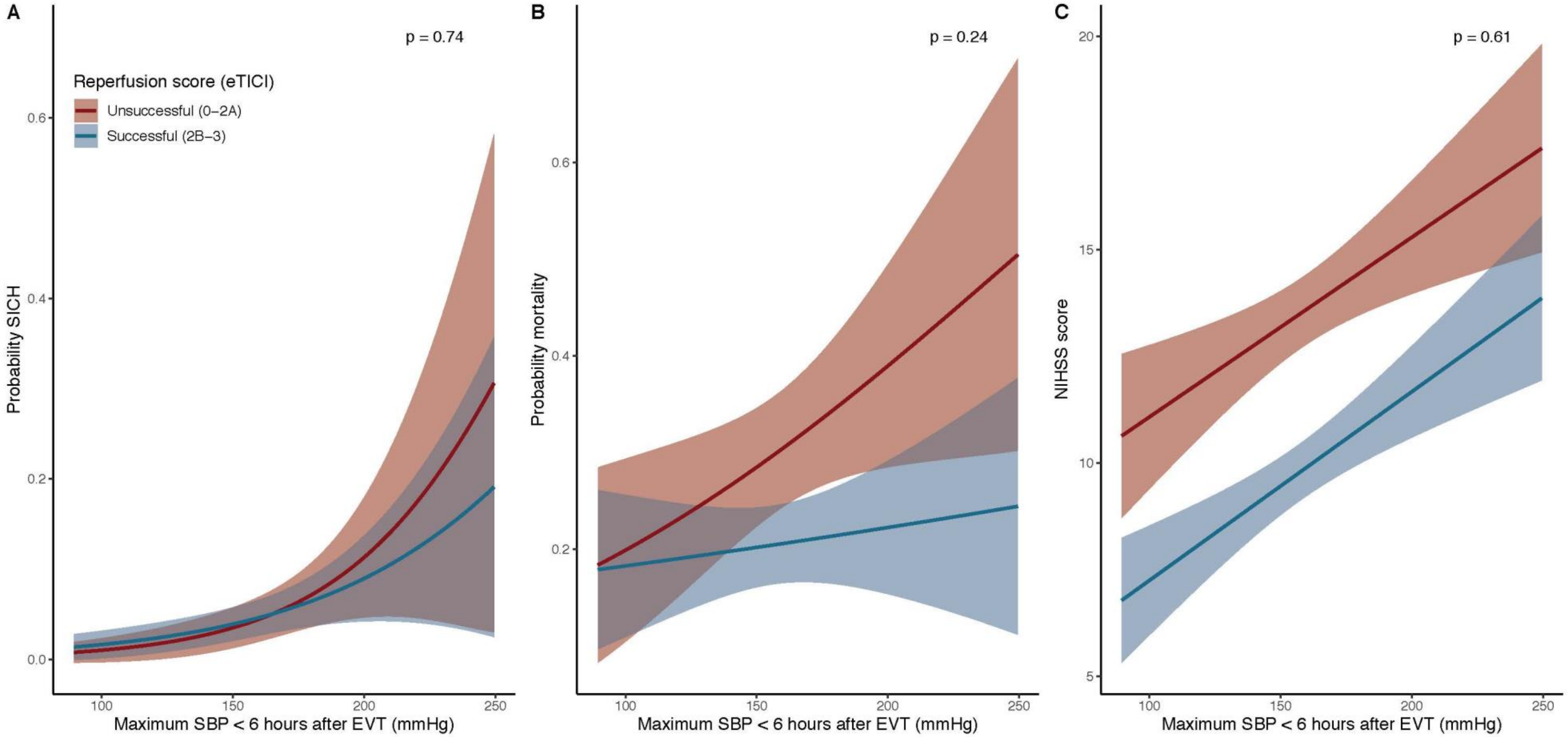

Figure II. Relationship of maximum SBP with probability of sICH, probability of mortality at 90 days and NIHSS at 24-48 hours following EVT.

The models include the following variables: maximum SBP, age, NIHSS at baseline, ASPECTS at baseline, history of hypertension, time between stroke onset to reperfusion, and an interaction term for maximum SBP*successful reperfusion. The figures depict the probability of symptomatic intracranial hemorrhage (A), the probability of mortality (B) and NIHSS score at 24-48 hours after EVT (C) with 95% confidence intervals, for each level of maximum SBP in the first 6 hours following EVT for successful and unsuccessful reperfusion separately and a p-value for interaction (maximum SBP*successful reperfusion).

Abbreviations: eTICI, extended Thrombolysis in Cerebral Infarction; EVT, endovascular treatment; mRS, modified Rankin Scale; NIHSS, National Institute of Health Stroke score; SBP, systolic blood pressure; sICH, symptomatic intracranial hemorrhage.

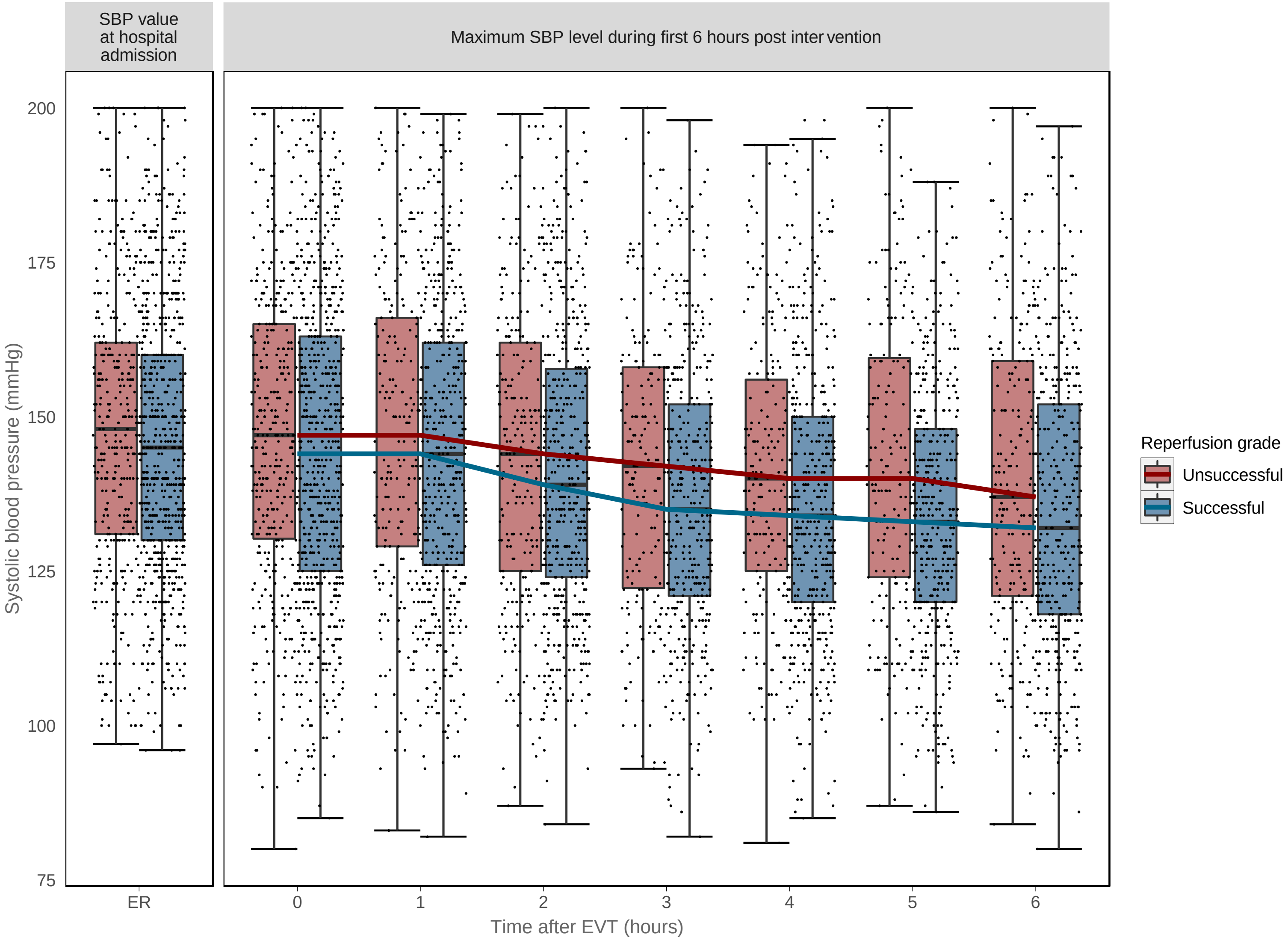

Figure III. Maximum SBP course in the first 6 hours following EVT for patients with successful reperfusion versus patient with unsuccessful reperfusion. Black dots represent individual SBP measurements. The boxplots indicate the interquartile ranges around the median which are reflected by the red (unsuccessful reperfusion) and blue (successful reperfusion) lines. EVT, endovascular treatment; SBP, systolic blood pressure.

MR CLEAN Registry Investigators – group authors

Executive committee
Diederik W.J. Dippel[1];Aad van der Lugt[2];Charles B.L.M. Majoie[3];Yvo B.W.E.M. Roos[4];Robert J. van Oostenbrugge[5];Wim H. van Zwam[6];Jelis Boiten[14];Jan Albert Vos[8]

Study coordinators
Ivo G.H. Jansen[3];Maxim J.H.L. Mulder[1,2];Robert- Jan B. Goldhoorn[5,6];Kars C.J. Compagne[2];Manon Kappelhof[3];Josje Brouwer[4];Sanne J. den Hartog[1,2,40];Wouter H. Hinsenveld [5,6]

Local principal investigators
Diederik W.J. Dippel[1];Bob Roozenbeek[1];Aad van der Lugt[2];Adriaan C.G.M. van Es[2];Charles B.L.M. Majoie[3];Yvo B.W.E.M. Roos[4];Bart J. Emmer[3];Jonathan M. Coutinho[4];Wouter J. Schonewille[7];Jan Albert Vos[8]; Marieke J.H. Wermer[9];Marianne A.A. van Walderveen[10];Julie Staals[5];Robert J. van Oostenbrugge[5];Wim H. van Zwam[6];Jeannette Hofmeijer[11];Jasper M. Martens[12];Geert J. Lycklama à Nijeholt[13];Jelis Boiten[14];Sebastiaan F. de Bruijn[15];Lukas C. van Dijk[16];H. Bart van der Worp[17];Rob H. Lo[18];Ewoud J. van Dijk[19];Hieronymus D. Boogaarts[20];J. de Vries[22];Paul L.M. de Kort[21]; Julia van Tuijl[21] ; Jo P. Peluso[26];Puck Fransen[22];Jan S.P. van den Berg[22];Boudewijn A.A.M. van Hasselt[23];Leo A.M. Aerden[24];René J. Dallinga[25];Maarten Uyttenboogaart[28];Omid Eschgi[29];Reinoud P.H. Bokkers[29];Tobien H.C.M.L. Schreuder[30];Roel J.J. Heijboer[31];Koos Keizer[32];Lonneke S.F. Yo[33];Heleen M. den Hertog[22];Emiel J.C. Sturm[35]; Paul J.A.M. Brouwers[34]

Imaging assessment committee
Charles B.L.M. Majoie[3](chair);Wim H. van Zwam[6];Aad van der Lugt[2];Geert J. Lycklama à Nijeholt[13];Marianne A.A. van Walderveen[10];Marieke E.S. Sprengers[3];Sjoerd F.M. Jenniskens[27];René van den Berg[3];Albert J. Yoo[38];Ludo F.M. Beenen[3];Alida A. Postma[6];Stefan D. Roosendaal[3];Bas F.W. van der Kallen[13];Ido R. van den Wijngaard[13];Adriaan C.G.M. van Es[2];Bart J. Emmer[,3];Jasper M. Martens[12]; Lonneke S.F. Yo[33];Jan Albert Vos[8]; Joost Bot[36], Pieter-Jan van Doormaal[2]; Anton Meijer[27];Elyas Ghariq[13]; Reinoud P.H. Bokkers[29];Marc P. van Proosdij[37];G. Menno Krietemeijer[33];Jo P. Peluso[26];Hieronymus D. Boogaarts[20];Rob Lo[18];Dick Gerrits[35];Wouter Dinkelaar[2]Auke P.A. Appelman[29];Bas Hammer[16];Sjoert Pegge[27];Anouk van der Hoorn[29];Saman Vinke[20].

Writing committee
Diederik W.J. Dippel[1](chair);Aad van der Lugt[2];Charles B.L.M. Majoie[3];Yvo B.W.E.M. Roos[4];Robert J. van Oostenbrugge[5];Wim H. van Zwam[6];Geert J. Lycklama à Nijeholt[13];Jelis Boiten[14];Jan Albert Vos[8];Wouter J. Schonewille[7];Jeannette Hofmeijer[11];Jasper M. Martens[12];H. Bart van der Worp[17];Rob H. Lo[18]

Adverse event committee
Robert J. van Oostenbrugge[5](chair);Jeannette Hofmeijer[11];H. Zwenneke Flach[23]

Trial methodologist
Hester F. Lingsma[40]

Research nurses / local trial coordinators
Naziha el Ghannouti[1];Martin Sterrenberg[1];Corina Puppels[7];Wilma Pellikaan[7];Rita Sprengers[4];Marjan Elfrink[11];Michelle Simons[11];Marjolein Vossers[12];Joke de Meris[14];Tamara

Vermeulen[14];Annet Geerlings[19];Gina van Vemde[22];Tiny Simons[30];Cathelijn van Rijswijk[21];Gert Messchendorp[28];Nynke Nicolaij[28];Hester Bongenaar[32];Karin Bodde[24];Sandra Kleijn[34];Jasmijn Lodico[34]; Hanneke Droste[34];Maureen Wollaert[5];Sabrina Verheesen[5];D. Jeurrissen[5];Erna Bos[9];Yvonne Drabbe[15];Michelle Sandiman[15];Nicoline Aaldering[11];Berber Zweedijk[17];Mostafa Khalilzada[15];Jocova Vervoort[21];Eva Ponjee[22];Sharon Romviel[19];Karin Kanselaar[19];Denn Barning[10].

PhD / Medical students:
Esmee Venema[40]; Vicky Chalos[1,40]; Ralph R. Geuskens[3]; Tim van Straaten[19];Saliha Ergezen[1]; Roger R.M. Harmsma[1]; Daan Muijres[1]; Anouk de Jong[1];Olvert A. Berkhemer[1,3,6];Anna M.M. Boers[3,39]; J. Huguet[3];P.F.C. Groot[3];Marieke A. Mens[3];Katinka R. van Kranendonk[3];Kilian M. Treurniet[3];Manon L. Tolhuisen[3,39];Heitor Alves[3];Annick J. Weterings[3],Eleonora L.F. Kirkels[3],Eva J.H.F. Voogd[11];Lieve M. Schupp[3];Sabine Collette[28,29];Adrien E.D. Groot[4];Natalie E. LeCouffe[4];Praneeta R. Konduri[39];Haryadi Prasetya[39];Nerea Arrarte-Terreros[39];Lucas A. Ramos[39].

List of affiliations
Department of Neurology[1], Radiology[2], Public Health[40], Erasmus MC University Medical Center;
Department of Radiology and Nuclear Medicine[3], Neurology[4], Biomedical Engineering & Physics[39], Amsterdam UMC, University of Amsterdam, Amsterdam;
Department of Neurology[5], Radiology[6], Maastricht University Medical Center and Cardiovascular Research Institute Maastricht (CARIM);
Department of Neurology[7], Radiology[8], Sint Antonius Hospital, Nieuwegein;
Department of Neurology[9], Radiology[10], Leiden University Medical Center;
Department of Neurology[11], Radiology[12], Rijnstate Hospital, Arnhem;
Department of Radiology[13], Neurology[14], Haaglanden MC, the Hague;
Department of Neurology[15], Radiology[16], HAGA Hospital, the Hague;
Department of Neurology[17], Radiology[18], University Medical Center Utrecht;
Department of Neurology[19], Neurosurgery[20], Radiology[27], Radboud University Medical Center, Nijmegen;
Department of Neurology[21], Radiology[26], Elisabeth-TweeSteden ziekenhuis, Tilburg;
Department of Neurology[22], Radiology[23], Isala Klinieken, Zwolle;
Department of Neurology[24], Radiology[25], Reinier de Graaf Gasthuis, Delft;
Department of Neurology[28], Radiology[29], University Medical Center Groningen;
Department of Neurology[30], Radiology[31], Atrium Medical Center, Heerlen;
Department of Neurology[32], Radiology[33], Catharina Hospital, Eindhoven;
Department of Neurology[34], Radiology[35], Medical Spectrum Twente, Enschede;
Department of Radiology[36], Amsterdam UMC, Vrije Universiteit van Amsterdam, Amsterdam;
Department of Radiology[37], Noordwest Ziekenhuisgroep, Alkmaar;
Department of Radiology[38], Texas Stroke Institute, Texas, United States of America.
